# Ehrlichiosis, Babesiosis, Anaplasmosis and Hepatozoonosis in Dogs from St. Kitts, West Indies

**DOI:** 10.1371/journal.pone.0053450

**Published:** 2013-01-15

**Authors:** Patrick J. Kelly, Chuanling Xu, Helene Lucas, Amanda Loftis, Jamie Abete, Frank Zeoli, Audrey Stevens, Kirsten Jaegersen, Kate Ackerson, April Gessner, Bernhard Kaltenboeck, Chengming Wang

**Affiliations:** 1 Ross University School of Veterinary Medicine, St. Kitts, West Indies; 2 Yangzhou University, Yangzhou, Jiangsu, People’s Republic of China; 3 College of Veterinary Medicine, Auburn University, Auburn, Alabama, United States of America; The Johns Hopkins University School of Medicine, United States of America

## Abstract

**Background:**

Although tick-borne diseases are important causes of morbidity and mortality in dogs in tropical areas, there is little information on the agents causing these infections in the Caribbean.

**Methodology:**

We used PCRs to test blood from a cross-section of dogs on St Kitts for *Ehrlichia* (*E*.) *canis*, *Babesia* (*B*.) spp., *Anaplasma* (*A*.) spp. and *Hepatozoon* (*H*.) spp. Antibodies against *E. canis* and *A*. *phagocytophilum/platys* were detected using commercial immunochromatography tests. Records of the dogs were examined retrospectively to obtain clinical and laboratory data.

**Principal findings:**

There was serological and/or PCR evidence of infections of dogs with *E. canis* (27%; 46/170), *Babesia* spp. (24%; 90/372) including *B. canis vogeli* (12%; 43/372) and *B. gibsoni* (10%; 36/372), *A. platys* (11%; 17/157) and *H. canis* (6%; 15/266). We could not identify the *Babesia* sp. detected in nine dogs. There was evidence of multiple infections with dual infections with *E. canis* and *B. canis vogeli* (8%; 14/179) or *B. gibsoni* (7%; 11/170) being the most common. There was agreement between immunochromatography and PCR test results for *E. canis* for 87% of dogs. Only 13% of exposed dogs had signs of a tick-borne disease and 38% had laboratory abnormalities. All 10 dogs presenting for a recheck after treatment of *E. canis* with doxycycline were apparently healthy although all remained seropositive and six still had laboratory abnormalities despite an average of two treatments with the most recent being around 12 months previously. Infections with *Babesia* spp. were also mainly subclinical with only 6% (4/67) showing clinical signs and 13% (9/67) having laboratory abnormalities. Similarly, animals with evidence of infections with *A. platys* and *H. canis* were largely apparently healthy with only occasional laboratory abnormalities.

**Conclusions:**

Dogs are commonly infected with tick-borne pathogens in the Caribbean with most having no clinical signs or laboratory abnormalities.

## Introduction

Tick-borne diseases are an important cause of morbidity and mortality in dogs worldwide with the brown dog tick, *Rhipicephalus* (*R.*) *sanguineus*, implicated as a vector of several disease agents including *A. platys*, *B. canis vogeli*, *B. gibsoni*, *E. canis*, spotted fever group (SFG) *Rickettsia* spp. and *H. canis* [1], [2], [3], [4]. Although there is some information on tick-borne infections of dogs in the Caribbean, each island has a unique immigration history and vector ecology and additional studies are needed to more fully understand infections in the region. Anecdotal reports suggested canine monocytic ehrlichiosis (CME) caused by *E. canis* occurs on Aruba, Puerto Rico, the Virgin Islands and Netherlands Antilles [5], [6], [7]. In early PCR studies, no DNA of SFG rickettsia was found in *R. sanguineus*, the most common tick of dogs in the Caribbean, from the islands of St Kitts (n = 52) [8] and Martinique (n = 11) [9]. But in a later study on St Kitts, Kelly et al. [10] found DNA of SFG rickettsiae in 6% of 143 *R. sanguineus*. They also found 15% had DNA of *Ehrlichia* spp., but none contained *Anaplasma* DNA. A PCR study on 73 dogs from Grenada showed them to be infected with *A. platys* (19%), *B. canis* (7%), *Bartonella* spp. (1%), *E. canis* (25%) and *H. canis* (7%) [11] while a study on 348 dogs from Trinidad showed dogs infected with *E. canis* (14%) and *B. canis vogeli* (7%) [12]. In a serosurvey of dogs from the Turks and Caicos Islands, 71% of feral dogs were positive for *E. canis* infection compared to 18% of pet dogs presenting to a veterinary clinic [13]. In the most recent study from the Caribbean, 13 thrombocytopenic dogs from St. Kitts were negative for *A. platys* and *Babesia* organisms by PCR [14].

To provide further information on tick-borne pathogens, we studied a large number of dogs from St Kitts to more precisely determine the prevalence of infections with tick-borne agents and obtain clinical data on infected animals.

## Materials and Methods

All work in this study was reviewed and approved by the Institutional Animal Care and Use Committee of the Ross University School of Veterinary Medicine.

### Dogs

The dogs were either patients at the Ross University School of Veterinary Medicine (RUSVM) Veterinary Teaching Hospital (VTH) or seen at the RUSVM Volunteers for Intercultural and Definitive Adventure (VIDA) clinics where dogs belonging to local people in underprivileged areas were provided with free basic veterinary care. Verbal or written consents from the owners of the dogs were received to use blood in this study.

### Blood Samples

Between December 2009 and November 2011, convenience samples of whole blood in ethylenediaminetetraacetic acid (EDTA) were obtained from the Diagnostic Laboratory of the RUSVM following their use for routine clinical laboratory testing including complete blood counts (CBC) and comprehensive biochemical profiles using VetScan HM5 and VetScan VS2 (Abaxis, Union City, CA, USA). In some cases, immunochromatography SNAP 4DX® or SNAP 3DX® (IDEXX Laboratories, Westbrook, Maine, USA) tests had also been requested and there were data on the presence of antibodies to *A. phagocytophilum/platys* and/or *E. canis*, respectively. Aliquots (200 µL) of the blood were frozen at −20°C until DNA was extracted as described below.

### Clinical Data

While no clinical data was available for VIDA dogs, records of the dogs presenting to the VTH were stored on an Avimark Veterinary Software System (McAllister Software Systems, LLC). These were reviewed to determine the reasons for presentation, the health status of the animal at the time of sample collection, preliminary diagnosis and laboratory data. A dog was regarded as having signs of a tick-borne disease if it had a history of tick infestation and/or clinical signs including fever, pallor, bleeding tendencies, lymphadenomegaly and splenomegaly. It was regarded as presenting for a routine health check if owners reported no significant abnormalities and the dog was apparently healthy when presented for routine vaccinations, a heart worm check or prior to elective procedures such as neutering or teeth cleaning. We reported there to be full data on a dog if there was a full history, complete physical exam and laboratory data including a CBC and routine biochemistry screen.

### DNA Extraction and Quantitative PCR

The High-Pure PCR Template Preparation Kit (Roche Molecular Biochemicals, Indianapolis, IN, USA) was used to extract total nucleic acid from canine whole blood as we previously described [15]. For each sample, 200 µl EDTA-whole blood was mixed with an equal volume of binding buffer [6 M guanidine-HCl, 10 mM urea, 20% (v/v) Triton X-100, 10 mM Tris-HCl, pH 4.4], and DNA samples were eluted in 150 µl elution buffer.

Quantitative PCR for *E. canis* 16S rRNA was performed on an Applied Biosystems 7500 Real-Time PCR System (Applied Biosystem, Foster City, CA, USA) as described previously [16]. Quantitative fluorescence resonance energy transfer (FRET)-PCRs for 16S or 18S rRNA gene of *Babesia* spp., *Anaplasma* spp. and *Hepatozoon* spp. were performed on a Roche LightCycler 2.0 PCR Instrument. Following the completion of FRET-PCR, the melting curve analysis for probes annealing to the PCR products was determined by monitoring the fluorescence from 37°C to 85°C with a temperature transition rate of 0.2°C per second [15]. The fluorescence ratio F4/F1 was analyzed, and the first derivatives of F4/F1 were evaluated to determine the probe melting temperature (*T*_m_)_._

**Figure 1:**
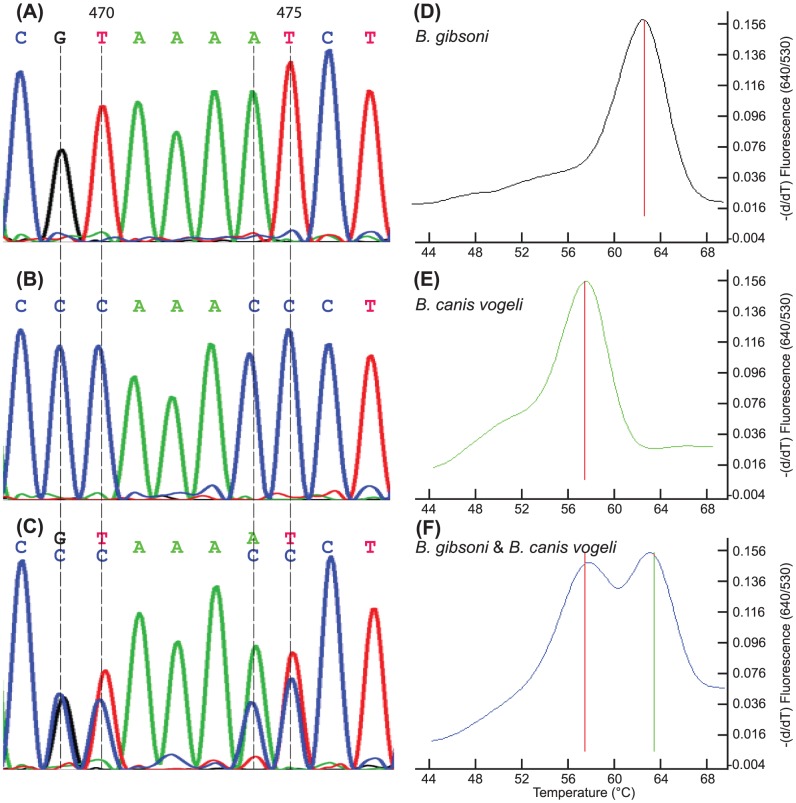
Sequencing results and melting curves of the *Babesia* spp. FRET-PCR. The upstream and downstream primers and the LCRed 640 probe of *Babesia* spp. FRET PCR are identical to the sequences of all *Babesia* spp. strains [15]. The fluorescein probe (5′-GACCC AAAAT CTCAC CAGAG TAACA ATTGG-6-FAM-3′) has two nucleotide mismatches to *B. gibsoni* (A, C; only a portion of the sequencing results were shown here), and three mismatches to *B. canis canis* and *B. canis vogeli* (B, C). Melting curves show the distinct *T*_m_ differences between *B. gibsoni* (∼63.5°C; D), and *B. canis vogeli* (∼57.0°C; E) as well as dual peaks indicating co-infections of *B. gibsoni* and *B. canis vogeli* (F).

The forward and reverse primers and the LCRed 640 probe used for *Babesia* qPCR had sequences common to all *Babesia* spp. strains [15] while the fluorescein probe (5′-GACCC AAAAT CTCAC CAGAG TAACA ATTGG-6-FAM-3′) had two nucleotide mismatches to *B. gibsoni* and three mismatches to *B. canis vogeli* (Figure 1). The *Anaplasma* qPCR primers (forward primer: 5′-GGT CGC AAG ACT AAA ACT CAA AGG AAT TGA CG-3′; reverse primer: 5′-TTA ACC CAA CAT CTC ACG ACA CGA GCT-3′) were designed to amplify portions of the 16S rRNA gene of both *Ehrlichia* spp. and *Anaplasma* spp., and specific probes were used to detect only *Anaplasma* spp. Two FRET probes [anchor probe: 5′-ACG CGA AAA ACC TTA CCA CTC CTT GAC–(6-FAM)-3′; quencher probe: 5′-(Bodipy 630/650)-TGG AGA TTA GAT CCT TCT TAA CGG AAG GG–(Phosphate)-3′] had identical sequences to *A. phagocytophilum* (AY055469, CP000235) and two nucleotide mismatches with *A. platys* (M82801, EF139459), but 8 with *E. ewingii* (M73227, U96436), 9 with *E. canis* (GU810149, EU178797) and 10 with *E. chaffeensis* (AF147752, CP000236). A nucleotide fragment representing part of the 16S rRNA gene of *A. phagocytopilum* was synthesized and inserted in the pIDTSMART cloningVector (Integrated DNA Technologies, Coralville, IA, USA). The plasmid was linearized with *Hind*III (Promega, Madison, WI, USA) and the restriction enzyme inactivated at 65°C for 20 min. DNA was quantified by the PicoGreen DNA fluorescence assay (Molecular Probes, Eugene, OR, USA) for preparation of quantitative standards in TE buffer.

The PCR for *Hepatozoon* spp. was performed as previously described [17]. High-resolution melting curve analysis was used to discriminate *H. canis* (*T*_m_ as ∼56°C) and *H. americanum* (*T*_m_ as ∼65°C).

The sensitivity and specificity of qPCRs for the detection of *E. canis*, *Babesia* spp. and *Hepatozoon* spp. were determined as described previously [15], [16], [17]. The size of the amplification products of the qPCRs were verified by gel electrophoresis and the amplicons were purified for DNA sequencing with a QIAquick PCR Purification Kit (Qiagen, Valencia, CA). Nucleotide sequencing was performed at the Genomic Sequencing Laboratory (Davis Sequencing, Davis, CA) using forward and antisense primers described here or in previous publications [15], [16], [17]. The specificity of *Anaplasma* qPCR was confirmed by DNA sequencing as well as testing on DNAs of *A. platys*, *A. phagocytophilum*, *E. canis*, *E. ewingii* and *E. chafeensis*. The *Anaplasma* PCR enabled the detection of 2 copies of 16S rRNA per PCR reaction which is equivalent to 150 copies of target DNA per ml of whole blood.

### Statistical Analysis

The presence or absence of pathogens in different groups was analyzed by chi-squared test. The PCR target numbers were logarithmically transformed for statistical analysis performed by Kruskal-Wallis non-parametric ANOVA.

## Results

### Samples Collected and Tests Performed

Blood samples were obtained from a total of 372 dogs, comprising 93 that presented to VIDA clinics and 279 that were seen at the VTH. The only analyses performed on the bloods from the VIDA clinics were PCRs for *Ehrlichia* spp., *Babesia* spp., *Anaplasma* spp. and *Hepatozoon* spp. These PCRs were also performed on all the 279 blood samples from dogs presenting to the VTH. Immunochromatography tests were only performed on bloods from dogs presented to the VTH with 63% (170/272) being tested for antibodies to *E. canis* and 58% (157/272) being tested for antibodies to *A. phagocytophilum/platys*. No serological tests were performed for *Babesia* spp or *Hepatozoon* spp.

Full data (clinical, CBC and routine biochemistry screen results) were found on 60% (159/279) of the VTH dogs, the majority of these dogs belonging to students or employees of the RUSVM. No clinical data was obtained on the dogs attending the VIDA clinics.

### Prevalence

The most common tick-borne agent in our study was *E. canis* with 24% (88/372) of the dogs having PCR and/or serological evidence of infection (Table 1). Nineteen-percent (69/372) were positive by PCR with the prevalence being significantly lower in dogs presenting to the VTH (11%; 31/279) compared to those attending the VIDA clinics (41%; 38/93) (P = 0.001). The average copy number in the *E. canis* PCR for dogs attending the VIDA clinics (148,362 copies of 16S rRNA/ml whole blood; n = 38) was significantly higher than that for the dogs presenting to the VTH (344 copies of 16S rRNA/ml whole blood; n = 31) (P = 0.02). Of the 125 owners of dogs presenting to the VTH that were questioned on tick control, most (75%; 94/125) reported the use of tick collars or spot-on preparations that local experience suggests are effective on the island. Although similar data was not available for owners of dogs presented to the free VIDA clinics, experience suggests that these dogs are commonly infested with ticks as the owners are unable to afford commercial tick control products.

**Table 1:** Prevalences of infections with tick-borne disease agents and numbers of dogs on which full data was available and their health status.

Tick-borne agent and test used to confirm exposure	Dogs positive	Dogs withfull data	Dogs with fulldata andapparentlyhealthy	Dogs withsigns of atick-bornedisease	Dogs with abnormal laboratory results
***Ehrlichia canis*** ** by +ve PCR**	19% (69/372)	29	76% (22/29)	ND	ND
**“ “ by +ve SNAP** *	24% (41/170)	39	59% (23/39)	ND	ND
**“ “ by +ve PCR and/or +ve SNAP** †	27% (46/170)	42	74% (31/42)	ND	ND
**Only ** ***E. canis*** ** by +ve SNAP and/or +ve PCR** †	17% (28/170)	24	79% (19/24)	**13% (3/24)**	**38% (9/24)**
**“ “ by +ve SNAP and +ve PCR** †	4% (6/170)	5	100% (5/5)	0%	40% (2/5)
**“ “ by −ve SNAP and +ve PCR** †	2% (3/170)	3	68% (2/3)	33% (1/3)	33% (1/3)
**“ “ by +ve SNAP and -ve PCR** †	11% (19/170)	16	75% (12/16)	13% (2/16)	38% (6/16)
***Babesia*** ** spp. by +ve PCR**	22% (90/372)	67	81% (54/67)	6% (4/67)	13% (9/67)
***B. vogeli*** ** by +ve PCR**	12% (43/372)	40	80% (32/40)	**10% (4/40)**	**13% (5/40)**
**Only ** ***B. vogeli*** ** by +ve PCR**	7% (25/372)	12	75% (8/12)	1% (1/12)	25% (3/12)
**“ “ and ** ***E. canis*** ** by PCR and/or SNAP**	8% (14/170)	8	63% (5/8)	25% (2/8)	13% (1/8)
***B. gibsoni*** ** by +ve PCR**	10% (36/372)	20	85% (17/20)	**20% (1/5)**	**20% (4/20)**
**Only ** ***B. gibsoni*** ** by +ve PCR**	6% (23/372)	12	75% (9/12)	0	17% (2/12)
**“ “ and ** ***E. canis*** ** by PCR and/or SNAP**	7% (11/170)	5	60% (3/5)	20% (1/5)	40% (2/5)
***Anaplasma platys*** ** by +ve PCR and/or +ve SNAP**	11% (17/157)	15	60% (9/15)	**7% (1/15)**	**0**
**“ “ by +ve PCR and +ve SNAP**	28% (3/17)	4	75% (3/4)	25% (1/4)	0
**“ “ by −ve PCR and +ve SNAP**	77% (13/17)	8	88% (7/8)	0	0
**“ “ by +ve PCR and –ve SNAP**	11% (1/9)	1	100% (1/1)	0	0
**Only ** ***A. platys*** ** by+ve SNAP and/or +ve PCR**	6% (9/157)	7	86% (6/7)	0	0
**“ “ by +ve SNAP and +ve PCR**	22% (2/9)	2	50% (2/4)	0	0
**“ “ by +ve SNAP and −ve PCR**	44% (4/9)	4	75% (3/4)	0	0
**“ “ by −ve SNAP and +ve PCR**	11% (1/9)	1	100% (1/1)	0	0
**“ “ and ** ***E. canis***	3% (5/157)	5	60% (3/5)	20% (1/5)	0
***Hepatozoon canis*** ** by PCR**	6% (15/266)	12	75% (8/12)	**0**	**2% (2/12)**
**Only ** ***H. canis*** ** by PCR**	3% (9/266)	6	83% (5/6)	0	0
**ditto and ** ***E. canis*** ** by PCR and/or SNAP**	2% (3/170)	2	100% (2/2)	0	100% (2/2)

* SNAP 3Dx® or SNAP 4 Dx®.

† For dogs tested by both PCR and SNAP.

ND – not determined.

The immunochromatography test results showed 19% (33/170) of the dogs tested from the VTH had antibodies reactive with *E. canis*. In the dogs that were tested by both PCR and the immunochromatography test, agreement between the two tests was present in 87% (148/170) of patients with both tests being negative for 84% (142/170) of the dogs and both tests being positive for 4% (6/170). Only the immunochromatography test was positive in 11% (19/170) of dogs and only the PCR was positive in 2% (3/170).

Our PCR study on *Babesia* spp. showed infections with the organisms were common (24%; 90/372) with the percentage of infected dogs that presented to the VTH (25%; 70/279) being similar to that for dogs attending the VIDA clinic (21%; 20/93). The fluorescent probe with its different level of mismatches with *Babesia* spp. and subspecies gave distinct *T*_m_ differences (Figure 1) which enabled us to determine and confirm by sequencing that there are two *Babesia* spp. on St Kitts, mainly *B. canis vogeli* (48%; 43/90) and *B. gibsoni* (40%; 36/90). Two dogs had DNA of both species as evidenced by dual melting point curves and 9 had unique melting temperatures which could not be accurately identified to the species level due to the limited length of the PCR amplicon available for sequencing. We obtained partial sequences (221 nucleotides of the 18S rRNA gene) for 27 of the dogs positive by PCR for *Babesia* spp. and, using the BLAST program (www.ncbi.nim.nih.gov/blast/), we found that the sequences of the *B. canis vogeli* (n = 12) from St Kitts were identical to one another, and to most of those reported from around the world including from Venezuela (DQ297390), Brazil (AY371194), Germany (AY371197) and the USA (AY072925). The sequences we obtained for the *B. gibsoni* from St. Kitts (n = 15) were also identical to one another, and to the vast majority of those reported from around the world, for example from widely disparate areas such as Australia (AY102164), Japan (AB118032), the USA (EU084677) and Germany (AF175300).

Immunochromatography tests for antibody to *A. phagocytophilum/A. platys* were performed on 157 blood samples with 17 giving positive reactions. The qPCR showed *A. platys* in 4 of these blood samples (28%) with a *T_m_* of 59.6°C which was substantially lower than the 69.6°C *T_m_* for the *A. phagocytophilum* sequence-containing plasmid standard. The presence of *A. platys* in the 4 positive amplicons was further confirmed by DNA sequence analysis.

Six percent (15/266) of the dogs tested by PCR had evidence of infection with *Hepatozoon* spp. Melting curve analysis indicated all were *H. canis* with a *T*_m_ of 58°C and this was confirmed by DNA sequencing of 4 amplicons.

Exposure of dogs to multiple agents was relatively common with 14% (51/372) having evidence of dual exposures. Those to *E. canis* and *B. canis vogeli* (35%; 14/51) and *E. canis* and *B. gibsoni* (22%; 11/51) were most common, while dual exposures to *A. platys* and *E. canis* (8%; 4/51) and *B. canis vogeli* and *H. canis* (2%; 1/51) were the least common. No dual exposures to *H. canis* and *A. platys* were seen while triple exposures to *E. canis*, *B. canis vogeli* and *A. platys* occurred in 4 dogs.

### Dogs with Full Data and Only Positive for *E. canis*, by PCR and/or Immunochromatography

#### a. PCR positive but immunochromatography test negative

All of the 3 dogs that were PCR positive and immunochromatography test negative had full data with 1 dog being suspected of having a tick-borne disease. This dog was anemic (RCC = 5.3×10^12^/L; N = 5.5–8.5×10^12^/L), thrombocytopenic (Plt = 186,000×10^9^/L; N = 200,000–500, 000×10^9^/L) and had hyperglobulinemia (Glob = 4.6 g/dL; N <4.5 g/dL). It had a higher copy number (174 copies of 16S rRNA/ml whole blood) than the other 2 dogs (1 copy of 16S rRNA/ml whole blood each) which presented for routine health checks and were apparently healthy on physical exam with no laboratory abnormalities.

#### b. PCR and immunochromatography test positive

Full data were available for 5 of the 6 dogs which were positive for *E. canis* only, by PCR and immunochromatography test. All were apparently healthy with 4 being presented for routine health checks and one for a recheck of previously diagnosed *E. canis* infection that had been treated a year previously with the recommended 10 mg/kg doxycycline given orally once daily for 3 weeks [18]. The laboratory data for 3 of the dogs was within normal limits but the dog being rechecked for *E. canis* had anemia (RCC = 5.3×10^12^/L), leukopenia (WCC = 4.2×10^9^/L; N = 6−17×10^9^/L) and hyperglobulinemia (Glob = 6.6 g/dL) and one other dog had anemia (RCC = 4.3×10^12^/L). The dogs with the laboratory abnormalities had higher copy numbers (1844 and 124 copies of 16S rRNA/ml whole blood) than those with normal values (1.1 and 1.2 copies of 16S rRNA/ml whole blood).

#### c. Immunochromatography test positive but PCR negative with no prior treatment

Of the 19 dogs that only had serological evidence of *E. canis* infection, we found full data on 7 that had not previously been treated with doxycycline. Two of the 4 dogs that presented because they were ill were suspected of having a tick-borne disease. One was collapsed and pancytopenic (WCC = 0.75 x 10^9^/L; RCC = 1.2 x 10^12^/L; Plt = 10,000 x 10^9^/L) with hypoplastic bone marrow, while the other had a microcytic anemia (RCC = 1.9 x 10^12^/L; MCV = 58 fL, N = 60–70 fL) suspected to be due to iron deficiency resulting from tick infestation. The other 2 ill dogs were presented for respiratory problems and the positive immunochromatography tests for *E. canis* infections were incidental findings when the dogs were tested for heartworms. The remaining 3 dogs were all apparently healthy on presentation for routine health checks although one had hyperglobulinemia (Glob = 6.0 g/dL).

#### d. Immunochromatography test positive but PCR negative with prior treatment

Of the 19 dogs that only had serological evidence of *E. canis* infection, we found full data on 9 that had been treated appropriately on at least one occasion [18]. Although all these dogs were apparently healthy on presentation for rechecking of their *E. canis* infection status, 5 dogs (56%; 5/9) still had laboratory abnormalities. One dog had leukopenia (WCC = 2.1×10^9^/L); two had hyperglobulinemia (Glob = 4.6 and 5.5 g/dL); one had thrombocytopenia (Plt = 10,000×10^9^/L) and hyperglobulinemia (Glob = 5.2 g/dL); and one had leukopenia (WCC = 2.2×10^12^/L), thrombocytopenia (Plt = 79,000×10^9^/L), elevated alkaline phosphatase (ALP = 215 IU/L; N = 20–150 IU/L), hypoalbuminemia (Alb = 1.9 g/dL, N >2.5 g/dL) and hyperglobulinemia (Glob = 5.3 g/dL). These dogs had all been treated once (n = 1), twice (n = 2) or three (n = 2) times before and had received their last treatment 5, 10, 25, 10 and 12 months previously, respectively. All but one dog with hyperglobulinemia had received 2 or 3 additional treatments beforehand. The dogs with no laboratory abnormalities had been treated 7, 8, 8 and 14 months previously with 3 of the dogs having had 2 to 3 treatments before.

#### e. PCR positive and copy numbers

None of the 7 dogs that had low copy numbers, arbitrarily defined as under 10 copies of 16S rRNA/ml whole blood, and full data had clinical signs of a tick-borne disease and only one had laboratory abnormalities, hyperglobulinemia (Glob = 5.2 g/dL) and thrombocytopenia (Plt = 8, 000×10^9^/L). Two of the 6 dogs with high copy numbers had clinical signs of a tick-borne disease and both these dogs, and three others with high copy numbers, had laboratory abnormalities (RCC = 1.7×10^12^/L, Alb = 1.8 g/dL and Glob = 8.1 g/dL; RCC = 3.0×10^12^/L and Plt = 3,000×10^9^/L; Plt = 44,000×10^9^/L; Plt = 14,000×10^9^/L; Glob = 5.7 g/dL).

### Clinical Data on Dogs Only Positive for *B. canis vogeli*

We obtained full data on 12 of the 25 dogs found by PCR to be infected with *B. canis vogeli* and having no evidence of other infections. Four of these were unwell, but only one was suspected to have a tick-borne disease based on clinical signs and pancytopenia (WCC = 4.8×10^9^/L; RCC = 4.9×10^12^/L; Plt = 9,000×10^9^/L). The remaining 3 unwell dogs had conditions not obviously related to *B. canis vogeli* infections, mainly heartworms and dog bite wounds. The 8 other dogs infected with *B. canis vogeli* were presented for routine health checks (75%; 8/12) with 3 having laboratory abnormalities; one was leukopenic (WCC = 4.4×10^9^/L), one was anemic (RCC = 4.84×10^12^/L) and one was thrombocytopenic (Plt = 187,000×10^9^/L). No *Babesia* parasites were reported in the CBCs of any of the dogs with *B. canis vogeli*.

### Clinical Data on Dogs Only Positive for *B. canis vogeli* and *E. canis*

We had full data on 8 of the 14 dogs that were coinfected with these two organisms. Two were PCR and immunochromatography test positive for *E. canis* and both of these dogs were suspected to have a tick-borne disease. Both dogs had pancytopenia (WCC = 0.72×10^9^/L, RCC = 1.3×10^12^/L, Plt = 6,000×10^9^/L; WCC = 3.7×10^9^/L, RCC = 2.6×10^12^/L, Plt = 3,000×10^9^/L) with the former one also being hypoalbuminemic (Alb = 1.6 g/dL) and having elevated alkaline phosphatase (ALP = 240 IU/L). One dog was only PCR positive for *E. canis* but was apparently healthy and had no abnormalities. Five dogs were only immunochromatography test positive including one dog presenting for emaciation which had normal laboratory values. The remaining 4 dogs were apparently healthy with 2 presenting for rechecks after treatment of *E. canis* infections. One of the latter had thrombocytopenia (Plt = 189,000×10^9^/L).

### Clinical Data on Dogs Only Positive for *B. gibsoni*

*Babesia gibsoni* was the second most common *Babesia* our PCR study identified, causing 40% (36/90) of the babesiosis cases and infecting 10% of the dogs we studied. Only two of the 36 (6%) dogs positive for *B. gibsoni* and for which breed data was available were pit bulls or their crosses although these represented 6% of the dogs for which breed data was recorded (12/205). Of the 23 dogs infected only with *B. gibsoni* we found full data on 12. No organisms were reported in the CBCs of the affected dogs. Twenty-five percent (3/12) were ill at presentation but did not have signs of a tick-borne disease. The remaining 75% (9/12) were presented for routine health checks with two having laboratory abnormalities; one had mild thrombocytopenia (189, 000×10^9^/L) and one had a mild anemia (RCC = 5.44×10^12^/L).

### Clinical Data on Dogs Only Positive for *B. gibsoni* and *E. canis*

Of the 11 dogs with evidence of infection with both organisms, we found full data on five (46%) which were all positive for *E. canis* by PCR and immunochromatography testing. Only one (25%) had clinical signs of a tick-borne disease and this dog also had thrombocytopenia (Plt = 11,000×10^9^/L). One dog had ingested a foreign body and was vomiting and three were presented for routine health checks. One of the latter had thrombocytopenia (Plt = 89,000×10^9^/L) and hyperglobulinemia (Glob = 6.2 g/dL) and one had anemia (RCC = 4.0×10^12^/L), thrombocytopenia (Plt = 129,000×10^9^/L), elevated alanine aminotransferase (Alt = 855 IU/L) and hyperglobulinemia (glob = 5.4 g/dL). None of the dogs had *Babesia* parasites reported in the CBC.

### Clinical Data on Dogs Only Positive for *B. gibsoni* and *B. canis vogeli*

Both of the dogs found to be infected with the two organisms by PCR were presented for routine health checks. None had parasites reported on the CBC with one being thrombocytopenic (Plt = 74,000×10^9^/L).

### Clinical Data on Dogs Only Positive for *A. platys*

As *Ixodes* spp., the vectors of *A. phagocytophilum*, are absent from St Kitts, we regarded animals positive in the immunochromatography test to have been exposed to *A. platys*. This was corroborated by our PCR studies in which we only identified *A. platys* on the island. We had full data for seven of the nine dogs with only evidence of *A. platys* infection. Five of the seropositive dogs, including two that were also PCR positive, and one dog seronegative but PCR positive, were presented for routine health checks and had no laboratory abnormalities. The final seropositive dog presented with neoplasia.

### Clinical Data on Dogs Only Positive for *A. platys* and *E. canis*

Five dogs had evidence of coinfection with *E. canis* with one, immunochromatography test positive for *E. canis* only, having signs of a tick-borne disease and thrombocytopenia (Plt = 12,000×10^9^/L) and hyperglobulinemia (Glob = 5.6 g/dL). Two dogs, immunochromatography test positive for *E. canis* and one immunochromatography test and PCR positive, were presented for routine health checks and were apparently healthy with no laboratory abnormalities. One dog positive by PCR for *A. platys* was presented with a fracture.

### Clinical Data on Dogs Only Positive for *A. platys, E. canis* and *B. canis vogeli*

Three dogs had evidence of infection with the three agents. Two dogs were apparently healthy although one had anemia (RCC = 1.4×10^12^/L), hypoalbuminemia (Alb = 1.8 g/dL) and hyperglobulinemia (Glob = 7.2 g/dL). The remaining dog had an abscess.

### Clinical Data on Dogs Only Positive for *H. canis*

We had full data on six of the nine dogs only positive for *H. canis*. Five of these were presented for routine health checks and were apparently healthy on presentation with normal laboratory results. One dog presented with neoplasia and the hepatozoonosis was an incidental finding.

### Clinical Data on Dogs Only Positive for *H. canis* and *E. canis*

Full data was available on two animals, one immunochromatography test and PCR positive for *E. canis* and the other immunochromatography test positive only. Both were presented for routine health checks and were apparently healthy with the first dog having leukopenia (WCC = 4.2×10^9^/L), anemia and hyperglobulinemia (Glob = 7.2 g/dL) and the second leukopenia (WCC = 2.2×10^9^/L).

### Clinical Data on Dogs Only Positive for *H. canis* and *Babesia* spp

Full data was not available on two dogs that were coinfected with *B. gibsoni* and one coinfected with *B. canis vogeli*.

## Discussion

Our study showed dogs on the island of St. Kitts in the Caribbean are commonly exposed to tick-borne organisms. Exposure to *E. canis* was the most common (24%) and this is consistent with reports that CME is very common on some other Caribbean islands from which data is available, Grenada (25%) [11], Turks and Caicos Islands (71%) [13] and Trinidad (14%) [12], and also around the world [19], [20], [21]. The vector of *E. canis* is the brown dog tick, *R. sanguineus*, which occurs commonly on St Kitts and is effectively the only tick found on dogs on the island [8], [14]. The significant difference in the prevalence of *E. canis* exposure in dogs presenting to the VTH and those presenting to VIDA was probably due to decreased infections resulting from more effective control of ticks in the former group. The higher copy numbers in the dogs presenting to VIDA might represent more acute infections being seen in these dogs [16] or it might result from different strains of *E. canis* circulating on the island [22].

We had full data on 24 of the 28 dogs which had immunochromatography test and/or PCR evidence of *E. canis* infection and no indication of other tick-borne diseases (Table 1). The finding of evidence of an *E. canis* infection in 48% (12/25) of these dogs was unexpected and occurred only because the dogs were having routine health checks which included testing for heartworms, the test kit for which includes the immunochromatography test for antibodies to *E. canis*. Although without signs of a tick-borne disease, five of the 12 dogs (42%) had laboratory abnormalities consistent with *E. canis* infection including thrombocytopenia, anemia, leukopenia and hyperglobulinemia [19], [23]. These abnormalities can affect the outcome of routine surgeries and testing for *E. canis* in dogs from the region should be considered before such procedures are performed.

The dogs found positive for *E. canis* in our study were treated according to the AVMA Consensus Statement on ehrlichial disease of small animals which was based on information available before its publication in 2002. It recommends dogs be given doxycycline if they are seropositive and/or PCR positive and there are compatible clinical and laboratory abnormalities [18]. Unfortunately, the best course of action for the 7 dogs we found that were immunochromatography test positive but normal clinically and by laboratory testing is not as straightforward. The AVMA Consensus Statement lists points for and against treating such dogs and the consensus is that treatment should be discussed with the owner and a decision made on the management course which is in the best interests of the dog [18]. A major consideration is that treatment of such animals will prevent them from progressing to the chronic phase of the disease (see below) where treatment is often unsuccessful and mortality is high [24].

In only 52% of cases (13/25) did clinicians specifically order the immunochromatography test for *E. canis*. These tests were requested for a dog with clinical signs of a tick-borne disease, two dogs presenting because of trauma which had inappropriately low platelet counts suspected to be due to *E. canis* infection, and 10 dogs being rechecked after appropriate treatment with doxycycline for *E. canis* infections [18]. The criteria for successful treatment are very clear in the AVMA Consensus Statement, mainly that dogs should become clinically normal, laboratory abnormalities resolve and the PCR and serology become negative [18]. Determining the success of treatment in practice, however, is generally not easy as laboratory abnormalities might take time to return to normal after treatment with thrombocytopenia usually taking 14 days and hyperglobulinemia taking up to nine months to normalize [18]. Further, although most dogs become seronegative by six to nine months after treatment, titers can remain elevated for years in some dogs [25], [26]. Also, although PCR is usually negative within two weeks of treatment [16] [27], [22], in some dogs it can remain intermittently positive after treatment [28]–[29].

All the dogs in our study failed to meet the criteria for successful treatment although three dogs with normal laboratory work were sampled seven to eight months post treatment, perhaps before the antibody titers had declined to non-detectable levels. Although immunochromatography tests are specific, sensitive and quick and simple to perform, they record any titer over about 1/100 as positive and it is not possible to determine if serum titers are declining [30], [31]. It should be noted, however, that the above dogs had all been treated two to three times previously, as had four of the six dogs that remained seropositive and still had laboratory abnormalities. Five of the latter dogs had been treated over nine months earlier and there should have been time for laboratory abnormalities and antibody titers to resolve.

Treatment failures with doxycycline have been reported in experimentally [28], [29], [32], [33], [34], [35] and naturally infected dogs [25], [22], [33]. There have been no carefully controlled longitudinal studies of dogs treated with doxycycline to determine the reasons for the apparent treatment failures. These have been summarized [19] and include antibiotic resistance, doxycycline administration problems, sequestration of *E. canis* and aberrant immune responses. Antibiotic resistance seems unlikely as *in vitro* susceptibility studies have shown that doxycycline has a bactericidal effect on *E. canis* at very low concentrations [36] and the susceptibility of the organism to the drug has been supported in many experimental infection studies [16], [27] [37]. A more likely reason for treatment failures is reinfection with *E. canis* as there appears to be no protective immune response in dogs after exposure and dogs can be reinfected following spontaneous or treatment clearance of infections [37], [38]. It is, then, very important that tick control measures are introduced to prevent reinfection post treatment. Although this is the policy of the VTH, owner compliance is not always complete so the authors regard this to be the most likely cause of the treatment failures we observed. This was most likely the case for the one recheck we found to be actively infected, that is PCR and immunochromatography test positive for *E. canis* and with laboratory abnormalities.

Our immunochromatography and PCR test results for *E. canis* were in agreement in a large percentage of dogs, supporting earlier reports of the sensitivity and specificity of immunochromatography tests [30], [31]. Similarly, our finding that PCR and immunochromatography tests were not always in agreement has also been reported previously, for example in dogs from Hong Kong [20], Colombia [21] and Grenada [11]. In these reports, 0% to 5% of dogs were immunochromatography test negative and PCR positive, which is similar to our findings of 2%. Similarly, about 17% (3% to 42%) were PCR negative but immunochromatography test positive, which is also comparable to our findings of 11%. These anomalous results probably arise because dogs are in different stages of infection.

Early experimental studies showed infections with *E. canis* have three phases, the acute, subclinical and chronic phases [38], [39], [40]. Dogs experimentally infected with *E. canis* usually become PCR positive within 4–10 days [16], [33], [41] but can take up to 28 days to develop reactive antibodies [42]. Therefore, animals that are PCR positive and immunochromatography test negative are considered to be in the acute phase of CME. Although experimental infections of dogs with *E. canis* cause clinical signs and laboratory changes in all dogs [16], [28], [32], [37], it is commonly stated that naturally acquired acute infections may be subclinical [18], [19], [23]. This seems to be based on wide experience of finding dogs infected with *E. canis* but with no history of compatible clinical signs. Although our numbers were small, the data supports this observation with 3 of the 4 dogs that appeared to be acutely infected, that is PCR positive and immunochromatography test negative, being clinically normal.

After the acute phase, dogs enter the subclinical phase of infection where they remain PCR positive and immunochromatography test positive [34], [43]. Although apparently healthy, they commonly have laboratory abnormalities, in particular thrombocytopenia (86%), anemia (57%), hyperglobulinemia (39%) and leukopenia (31%), singly or in combinations [18], [19], [43], [44]. In our study we found four dogs with full data in this stage of the disease. All were apparently healthy with only one having a laboratory abnormality which provides further substantiation that many natural *E. canis* infections are subclinical [24], [51]. As in acute infections, deciding on treatment for animals with no laboratory abnormalities is difficult.

Following the subclinical phase of the disease some dogs will have spontaneous resolution of infections [34], [35], [37]. Such dogs have no history of treatment but become PCR negative with laboratory abnormalities and antibody titers declining slowly over six to nine months. In our study, 24% (6/25) of the dogs with evidence of *E. canis* infection were immunochromatography test positive, PCR negative and with no clinical or laboratory abnormalities. These dogs could, then, have been undergoing spontaneous resolution of their infections. This is consistent with the proportion of dogs described to have spontaneous resolution in experimental infections (30–70%) [34], [35], [37], and supports a previous report that spontaneous recovery also occurs in naturally infected dogs [45].

In rare instances, following the subclinical phase of the disease, a severe and life-threatening chronic phase of the disease may develop. In this phase, dogs are usually seropositive and have clinical signs including anorexia, weight loss, emaciation, bleeding tendencies and severe pancytopenia with hypoplastic bone marrow [24], [39]. Our study confirmed that the chronic phase of the disease is uncommon as, from all the dogs on which we had full data, we could only identify 3 (7%; 3/42) that were probably in this phase of the disease. One was seropositive for *E. canis* and 2 were immunochromatography and PCR test positive and coinfected with *B. canis vogeli*. We found only one report describing the PCR status of dogs in the chronic phase of the disease and this showed most (13/19) had bone marrow positive by PCR for *E. canis* [24]. Our finding of 2/3 dogs with positive PCRs of whole blood suggests either sample can be used for confirming *E. canis* infection and further demonstrates that detecting *E. canis* in pancytopenic dogs in the chronic phase of the disease can be difficult.

Canine babesiosis is caused by the small *B. gibsoni* and three strains of the large *B. canis* namely *B. canis canis*, *B.canis vogeli* and *B. canis rossi*. The results of a previous study on St Kitts indicated there was no babesiosis on the island as DNA of *Babesia* spp. was not found in 13 dogs from the island [14]. The primers used in that study were developed to detect US strains [46] but should have detected the *Babesia* we found as our sequencing data showed them to be closely related to strains in the US and others around the world. A more likely explanation for the inability to find *Babesia* sp. in the above study is the fact that the only dogs tested were those that were apparently healthy, thrombocytopenic and seronegative for *E. canis*. We found that only a small percentage of such dogs infected with *Babesia* spp. have thrombocytopenia (11%; 2/17) suggesting the above study's sample group may not have been ideal for detecting *Babesia* spp.

We failed to identify *B. canis canis* and *B. canis rossi* on the island probably because their vectors, *Dermacentor* spp. [47] and *Haemaphysalis elliptica* [48], respectively, are not present on St Kitts. The brown dog tick, *R. sanguineus*, is the vector of *B. canis vogeli* [47] and is a very common tick on dogs on the island [8], [14]. It is not surprising that this was the principal species we encountered causing 48% (43/90) of the *Babesia* spp. infections. *Babesia canis vogeli* is found widely in the tropical and subtropical regions of most continents and has been reported from the Caribbean previously with 7% of dogs in both Grenada [11] and Trinidad [12] being infected. Organisms were not seen in 187 blood smears from dogs on the Turks and Caicos Islands [13] but our laboratory also did not report parasites in routine blood films, even though we had a high prevalence of infection by PCR. In the US, prevalences vary from 4–59% with most infections being subclinical and most infected dogs being subclinical carriers [49]. This was also the situation on St Kitts where we found only a single case (2%) of *B. canis vogeli* causing acute clinical signs in the 43 infected dogs. The infections in the remaining dogs were incidental findings when the dogs presented for routine health checks or for other conditions and only occasionally were mild laboratory changes seen.

*B. gibsoni* is found in northern Africa, southern Asia, Australia, Europe and the USA [47]. We now report it from the Caribbean. It would appear, however, that the organism is not widely distributed in the region as recent studies on Grenada and Trinidad failed to identify *B. gibsoni* [11], [12]. There are a number of suspected vectors of *B. gibsoni*, mainly *R. sanguineus*, *H. longicornis*, *H. leachii*, *H. bispinosa* and *D. variabilis* [49]. The fact that the only tick on this list found on St Kitts is *R. sanguineus* provides further evidence for it being a significant vector of *B. gibsoni*. High prevalence of infection in pit bulls in the US has led to suggestions that this breed is more susceptible and fighting might be a means of transmission of *B. gibsoni* [50], [51]. This appeared not to be the case in our study, however, as pit bulls and their crosses comprised 6% of the dogs we studied yet they comprised only 4% (2/46) of the dogs we found infected with *B. gibsoni*.

Our sequencing data showed the *Babesia* spp. we found on St Kitts were identical to one another and those from the US and around the world and did not enable us to determine their geographic origin. We do know, however, that students from the US commonly bring their dogs with them when attending universities on the island. It is likely that some of these dogs could have been subclinically infected and a source of infection for the *R. sanguineus* vectors and naïve dogs on the island. Recently, new *Babesia* spp. of dogs have been described in the United States including *Babesia* sp. (Coco) [52] and *B*. *conradae* [53], and animal health authorities on Caribbean islands might be advised to request PCR screening of visiting dog for *Babesia* to prevent introductions of further species. We were unable to identify the *Babesia* sp. in 9 of the dogs we studied although the sequence data we obtained allowed us to determine they were not the above organisms or *Theileria equi* and *B. caballi*, species known to infect horses on the island and dogs in Europe [54], [55]. Further work is underway in our laboratory to further characterize these *Babesia* spp.

*Babesia gibsoni* is generally regarded as a pathogenic species causing acute signs including fever, pallor, anorexia and weight loss [56]. There is also a chronic form that is seen frequently in the US [49] and has the above signs and splenomegaly, hepatomegaly and lymphadenopathy. Asymptomatic infections have also been reported [46] [50] [55] and this was our experience on St Kitts with over half of the dogs for which we had full data having no clinical or laboratory abnormalities. When they were present, they were mild and consistent with those previously reported [56].

Although the immunochromatography test detects antibodies to both *A. phagocytophilum* and *A. platys*, the vectors for the former, *Ixodes* spp., is not present on St Kitts and this, together with our *T_m_* and sequencing results, demonstrated that *A. platys* was the organism we detected in our study dogs. The US strains of *A. platys*, the agent of infectious canine thrombocytopenia, generally cause subclinical infections in dogs while those from elsewhere are more likely to cause clinical signs including fever, lymphadenopathy and bleeding disorders [57], [58], [59]. The *A. platys* we found on St Kitts seems to be most similar to that in the US. Most of the dogs we found with evidence of infection with the organism were apparently healthy with no laboratory abnormalities. This included three dogs that were PCR positive and thus most likely with active infections.

We detected *H. canis* infections in 4% of the dogs sampled but, as is the case elsewhere, the organism appears to be of low pathogenicity [3]. Most of the infected dogs on St Kitts had no clinical or laboratory signs. Laboratory abnormalities were seen in dogs coinfected with *E. canis* but these were most likely due to the *E. canis* infection.

In conclusion, our data provides further evidence that dogs in the Caribbean are commonly infected with tick-borne agents and veterinarians treating these animals should have a high index of suspicion of infection. Further, dogs from the Caribbean are commonly subclinical carriers of tick-borne organisms and animal health workers dealing with the importation of these dogs into non-endemic areas should consider appropriate testing and quarantine.
